# The fate of minor alkali elements in the chemical evolution of salt lakes

**DOI:** 10.1186/1746-1448-7-2

**Published:** 2011-10-12

**Authors:** Rebecca A Witherow, W Berry Lyons

**Affiliations:** 1 School of Earth Sciences, The Ohio State University, Columbus, Ohio, USA; 2 Byrd Polar Research Center, The Ohio State University, Columbus, Ohio, USA; 3 Idaho Dept. of Environmental Quality, Coeur d'Alene, Idaho, USA

## Abstract

Alkaline earth elements and alkali metals (Mg, Ca, Na and K) play an important role in the geochemical evolution of saline lakes as the final brine type is defined by the abundance of these elements. The role of major ions in brine evolution has been studied in great detail, but little has been done to investigate the behaviour of minor alkali elements in these systems despite their similar chemical affinities to the major cations. We have examined three major anionic brine types, chloride, sulphate, and bicarbonate-carbonate, in fifteen lakes in North America and Antarctica to determine the geochemical behaviour of lithium, rubidium, strontium, and barium. Lithium and rubidium are largely conservative in all water types, and their concentrations are the result of long-term solute input and concentration through evaporation and/or sublimation. Strontium and barium behaviours vary with anionic brine type. Strontium can be removed in sulphate and carbonate-rich lakes by the precipitation of carbonate minerals. Barium may be removed in chloride and sulphate brines by either the precipitation of barite and perhaps biological uptake.

## Background

The ultimate chemistry of a saline, closed-basin lake is determined by the initial composition of precipitation, the weathering reactions between dilute inflow water and lithology, and evapoconcentration (or sublimation) of the lake water [1]. The chemical pathways of evolving brines, termed geochemical divides, are determined very early by the initial chemical composition [1]. During mineral precipitation from a brine, the less abundant ion of the mineral pair of ions will become drastically depleted compared to the other [1].

**Figure 1:**
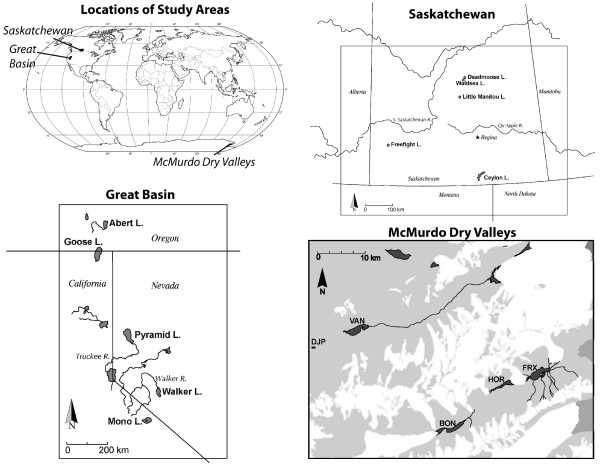
**Study Locations**. The locations of the sites examined in this study.

Modelling of closed-basin lakes has focused on the major elements of most natural waters: Ca, Na, K, Mg, Cl, SO_4_, and carbonate alkalinity (HCO_3 _+ CO_3_) and the simple salts that these ions produced during evaporation [1-3]. This research was fundamental in illustrating how the geochemistry of a lake can result from dilute inflow water with a composition unlike the final brine. It is clear from these models that the abundance of major alkali elements, Ca, Na, K, and Mg, is key in determining the geochemical pathways involved in brine formation, yet there has been little work to extend the modelling efforts to aid in the prediction of minor and trace metal behaviour during brine formation. In this paper, we have made an attempt to understand minor alkali metal and alkaline earth behaviour in different brine types in order to extend the earlier efforts on major cations. We have analysed water samples from brackish and saline lakes in the Great Basin of the United States, Saskatchewan, and the McMurdo Dry Valleys of Antarctica (Figure 1) and used these data in the geochemical model, PHREEQ, to illustrate the potential removal mechanisms that are occurring in closed-basin lakes.

### Site descriptions

#### McMurdo Dry Valleys

The McMurdo Dry Valleys (MCM) are the largest ice-free areas in Antarctica (approximately 4800 km^2^) (Figure 1) [4]. The MCM (≈78° S) are a polar desert classified by extremely low average annual temperatures (-16°C to -21°C) and high aridity (< 10 cm snowfall per year) [5]. Even in these harsh conditions, perennially ice-covered lakes and hypersaline ponds exist in Taylor Valley (Lakes Fryxell, Hoare, and Bonney) and in Wright Valley (Lake Vanda and Don Juan Pond) [6].

The ephemeral streams in the MCM flow between 6-10 weeks during the austral summer from November to January and flow is highly variable both daily and seasonally [5,7]. The water in these ephemeral streams is derived from glacial melt and it is the sole source of water to closed-basin lakes of the MCM. The only means of maintaining hydrological balance in the lakes is by sublimation of the perennial ice covers, which are replenished by the freezing of lake water on the bottom of the ice [8].

Previous work on the MCM lakes has shown that despite the great range in salinity (TDS = 40-645,000 mg/L), all of the MCM lakes have Cl as the major anion [9,10]. The glacial melt inflow is enriched in marine aerosols, and the dissolution of aeolian-deposited marine salts contributes to enrichment in chloride [10,11]. Sodium is the dominant cation in the Taylor Valley Lakes (Bonney, Fryxell, and Hoare) and Ca is the dominant cation in Lake Vanda's hypolimnion and Don Juan Pond [12,13].

#### Great Basin lakes

The geochemistry of Great Basin lakes has been studied in great detail and was key in developing the brine evolution model [14]. Mono Lake, Pyramid Lake, and Walker Lake, are the remnants of Pleistocene Lake Lahontan (Figure 1), and are characterized by relatively high concentrations of carbonate and bicarbonate. As these lakes evaporated, calcium and magnesium were removed as sulphate and carbonate minerals, drastically depleting the lakes of alkaline earth elements [14]. Lake Abert, in southern Oregon, and Goose Lake, on the border between Oregon and California, are remnants of Pleistocene Lake Chewaucan (Figure 1). Mean annual temperatures in the northern reaches of the Great Basin range from 23° to 32°C, and the majority of water entering these lakes comes from seasonal snowmelt in the surrounding mountains [15]. Lake Abert is a Na-Cl-CO_3 _brine with TDS ranging from 18,700-95,000 mg/L. Because it has been periodically refreshed by overflow, Goose Lake, a Na-HCO_3 _brine, is much fresher with TDS ranging from 600 to 2,700 mg/L. The wide range in salinities in these lakes is due to climatic conditions and anthropogenic activities which dictate the balance of evaporation and inflow. In Abert and Goose Lakes, it has been suggested that carbonates and silicates are being removed by inorganic precipitation and biogenic processes [15].

#### Canadian Prairie lakes

It is estimated that the Great Plains of Canada have between one and ten million saline lakes (Figure 1) [16]. The lakes are young (< 10,000 years old), and most are remnants of larger extinct proglacial lakes such as Lake Regina, Lake Hind, Lake Saskatchewan, and Lake Agassiz [16]. The area is characterized as a cold, semi-arid climate, and although the mean annual temperature is 3°C, the low humidity, high winds, and warm summer temperatures are conducive to high evaporation rates [17]. During the cold, clear winter months, many lakes form an ice cover, and some lakes have undergone periodic desiccation and refilling [16,18].

The lakes show a wide range in salinity from brackish (TDS = 1,000-5,000 mg/L) to saline (TDS > 5,000 mg/L) [16]. Sulphate along with Mg, Ca, and Cl demonstrate considerable range and relative concentrations between lakes [17]. Ceylon Lake, Freefight Lake, and Waldsea Lake are dominated by sulphate and Deadmoose Lake and Little Manitou Lake are mixed SO_4_-Cl brines. In all of the lakes, sodium is the dominant cation indicating that either the inflow waters were highly depleted in calcium and magnesium and/or that these cations have been removed by the precipitation salts. The larger, perennial lakes are chemically and thermally stratified with complex mineral precipitation/dissolution profiles [18]. The lakes precipitate calcite and high magnesium calcite and periodic whiting has been observed in Deadmoose and Waldsea Lakes [16,17].

All of these lakes in this study were selected because they are brackish to hypersaline or, in the case of MCM lakes, their hypolimnia are brackish or hypersaline. Because their major anion concentrations vary greatly, the comparison of these very different closed-basin systems provide an excellent test of the importance of anionic composition on minor-element distribution and potential mineral precipitation.

## Results

### Concentrations: Major elements

The salinities, as TDS, of the Canadian lakes ranged from 2.7% (Deadmoose Lake) to 23%. (Ceylon Lake) (Additional file 1). The salinities of the lakes in the Great Basin ranged from 0.4% (Pyramid Lake) to 12% (Abert Lake). The range of the salinity of the MCM lakes was 0.003% to 44% with the lowest values in the surface water of Lake Hoare and the highest salinity in Don Juan Pond.

Sodium was the dominant cation in all of the lakes in the MCM with the exception the hypolimnion of Lake Vanda and Don Juan Pond, where Ca was dominant. Na concentrations ranged from 20.3 mg/L in the surface water of Lake Hoare to 48,500 mg/L at 35 m in E. Lake Bonney. All of the McMurdo Dry Valley lakes/ponds had chloride as the dominant anion. The water at 4 m in Lake Hoare had the lowest concentrations of Cl (33.5 mg/L), and Don Juan Pond had the highest Cl concentrations of all lakes in the MCM (283,000 mg/L). Na was the dominant cation in the Canadian lakes where concentrations ranged from 2,160 mg/L in Waldsea Lake to 196,000 mg/L in Ceylon Lake. Sulphate concentrations ranged from 14,800 mg/L in Deadmoose Lake to 196,000 mg/L in Ceylon Lake. The highest concentration of Cl was in Little Manitou Lake (21,300 mg/L), and the lowest concentration occurred in Freefight Lake (4,190 mg/L). The lakes in the Great Basin had Na as the dominant cation. Mono Lake had the highest Ca concentrations, 3.7 mg/L. Ca was below the detection limit in Abert Lake; this has been shown in previous work as well [19]. Mono and Goose Lakes were carbonate-rich, Pyramid Lake was chloride-rich, and Abert and Walker Lakes were enriched in both HCO_3_+CO_3 _and Cl. The pH values in the MCM lakes were generally lower than the lakes in Saskatchewan, which were lower than the alkaline lakes of the Great Basin.

**Figure 2:**
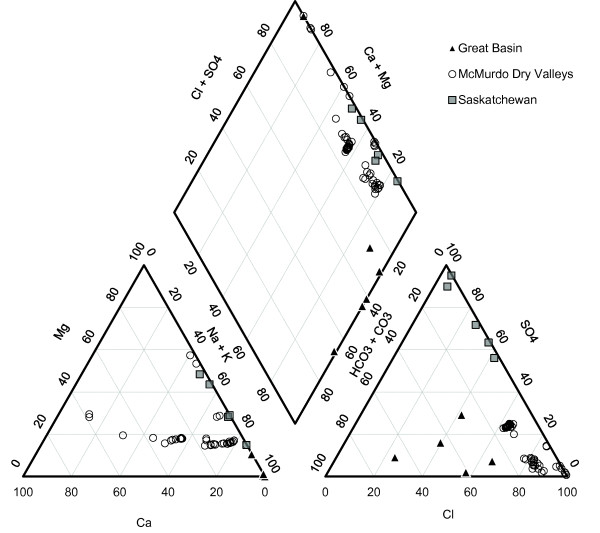
**Piper diagram of the lakes in this study**. Concentrations are in equivalent %.

The major element geochemistry of all these lakes are depicted in Piper diagrams in Figure 2. (We have utilized Piper diagrams to visually demonstrate the difference in the major ion geochemistry of the three different lakes settings and to show the impact of the changes in anion dominance on the major cation geochemistry). Of all the lakes studied, the Great Basin lakes had the highest concentrations of HCO_3 _+ CO_3_, and the Saskatchewan lakes had the highest concentrations of SO_4_. The hypersaline waters of the MCM had the highest Cl, Ca, and Mg concentrations, and their surface, fresher waters had the lowest. So, for simplicity we shall term the Great Basin lakes, the "carbonate lakes," the Saskatchewan lakes, the "sulphate lakes," and the McMurdo Dry Valley lakes, the "chloride lakes."

### Concentrations: Minor elements

**Figure 3:**
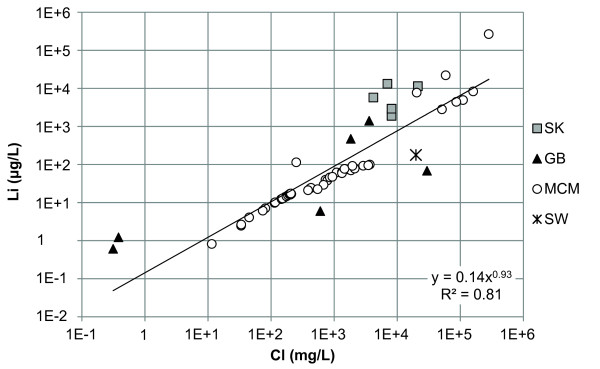
**Chloride and lithium concentrations in the lakes in this study**. SK = Saskatchewan, GB = Great Basin, MCM = McMurdo Dry Valleys, SW = seawater [61].

**Figure 4:**
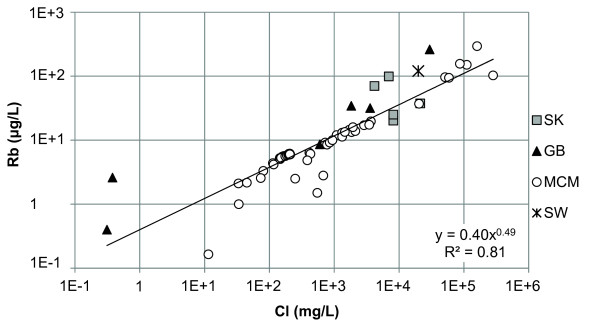
**Chloride and rubidium concentrations in the lakes in this study**. SK = Saskatchewan, GB = Great Basin, MCM = McMurdo Dry Valleys, SW = seawater [61].

**Figure 5:**
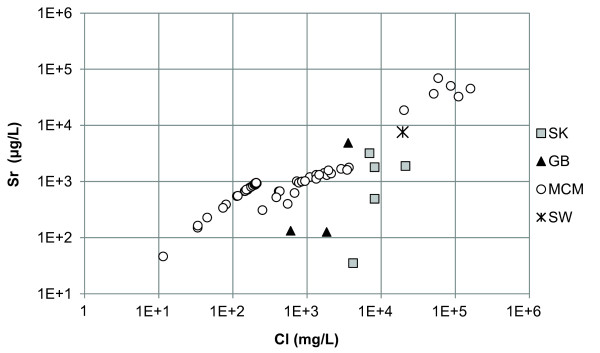
**Chloride and strontium concentrations in the lakes in this study**. SK = Saskatchewan, GB = Great Basin, MCM = McMurdo Dry Valleys, SW = seawater [61].

**Figure 6:**
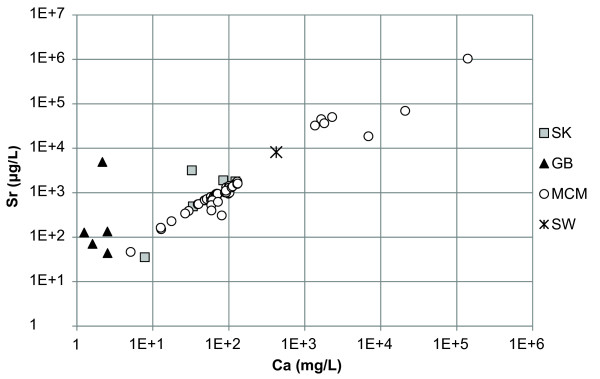
**Calcium and strontium concentrations in the lakes in this study**. SK = Saskatchewan, GB = Great Basin, MCM = McMurdo Dry Valleys, SW = seawater [61].

**Figure 7:**
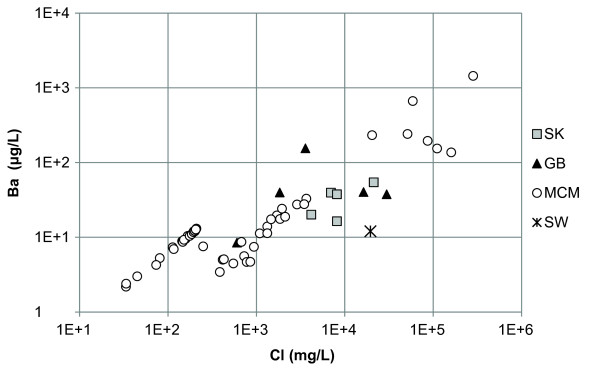
**Chloride and barium concentrations in the lakes in this study**. SK = Saskatchewan, GB = Great Basin, MCM = McMurdo Dry Valleys, SW = seawater [61].

Of the minor elements, Li, Rb, Sr, and Ba, the Canadian and Great Basin lakes had Li in the highest concentration except Walker Lake which had Sr concentrations higher than Li (Additional file 2). Lake Hoare, Lake Fryxell, and West Lake Bonney are described by high concentrations of Sr. Lake Vanda, Don Juan Pond, and the monimolimnion of East Lake Bonney had high concentrations of Li and Sr. In general Li and Rb demonstrated positive relationships with Cl (Figures 3 and 4). Strontium had a strong relationship with Ca but a poor relationship with Cl (Figure 5 and 6). In general, Ba and Sr do not correlate well with any major or minor elements including Cl (Figures 3, 4, 5 and 7). The strong correlations for Li and Rb with Cl suggest conservative behavior of these cations through the evapoconcentration/sublimation process. Note that the seawater cation and Cl values are shown on these plots for reference. There is an apparent relationship between Ba and Cl at the lower salinities but this relationship is different among the lakes (Figure 7).

Don Juan Pond had the highest Li (2.65 × 10^5 ^μg/L), Sr (1.04 × 10^6 ^μg/L), and Ba (1.4 × 10^3 ^μg/L) concentrations of all the MCM lakes (Additional file 2). The highest Rb concentration (294 μg/L) was found 35 m below the surface of E. Lake Bonney. Of all the MCM lakes, Lake Hoare, the freshest lake in this study, had the lowest Li (2.47 μg/L), Rb (2.11 μg/L), Sr (150 μg/L), and Ba (1 μg/L) concentrations. These occurred at 4 m depth. In all of the MCM lakes, Li, Rb, and Sr increased in concentration with depth. Barium concentrations increased with depth in Lakes Hoare, Fryxell, and Vanda, but the hypolimnetic waters of both lobes of Lake Bonney had concentrations lower than samples from above the chemocline. In the Saskatchewan lakes, Ceylon Lake had the highest concentrations of Li (1.31 × 10^4 ^μg/L), Rb (98.8 μg/L), and Sr (3.18 × 10^3 ^μg/L). Little Manitou Lake had the highest concentration of Ba (54 μg/L). Deadmoose Lake had the lowest concentrations of Li (1.9 × 10^3 ^μg/L), Rb (20.2 μg/L), and Ba (16 μg/L), and Freefight Lake had the lowest Sr concentration (35.1 μg/L). In the Great Basin, Mono Lake had the highest concentrations of Li (9.43 × 10^3 ^μg/L) and Rb (1.63 × 10^3 ^μg/L) and Goose Lake had the lowest (5.90 μg/L and 8.60 μg/L, respectively). Goose Lake also had the lowest Ba concentration (8 μg/L). Walker Lake had the highest concentration of Ba (160 μg/L) and Sr (4.85 × 10^3 ^μg/L). The Sr concentrations of Mono Lake and Abert Lake were below our detection limit of 0.05 μg/L.

### PHREEQ modelling

In order to establish if the geochemistry of the salt lakes in our study is controlled by salt formation, we have used the computer program PHREEQ to determine mineral saturation indices within the lake waters (see Methods for details) (Additional file 3). Although the model calculates the saturation states of a large suite of minerals, we have focused on only the simple binary salts of the elements of interest which include: anhydrite (CaSO_4_), aragonite (CaCO_3_), barite (BaSO_4_), calcite (CaCO_3_), celestite (SrSO_4_), magnesite (MgCO_3_), strontianite (SrCO_3_) and witherite (BaCO_3_). The hydrated salts, gypsum (CaSO_4_·2H_2_O) and mirabilite (Na_2_SO_4_**·**10H_2_O) play a critical role in the evolution of many saline waters and are also included in our calculations. The MCM calculations were performed using *in-situ *measured temperatures. Because we did not have the temperatures of the samples when they were obtained, we calculated saturation indices for the Great Basin and Saskatchewan lakes at two end member temperatures, 0°C and 20°C in order to examine how seasonal temperature variations might affect mineral precipitation within the lakes.

### Modelling results

The saturation indices for all the lakes are tabulated in Table 1. The Canadian Lakes were all supersaturated with respect to magnesite at 0°C and 20°C (Table 1). Waldsea Lake was supersaturated with aragonite, barite, and calcite at 0°C and 20°C. Little Manitou and Deadmoose Lakes were supersaturated with barite at 0°C and 20°C, supersaturated with respect to calcite at 20°C, and were in near equilibrium with aragonite at 20°C. At 0°C, Little Manitou and Deadmoose Lakes were undersaturated with calcite and aragonite. With respect to barite, Freefight and Ceylon Lakes were near equilibrium at 20°C and supersaturated at 0°C. At 0°C, Ceylon Lake was the only lake in this study to be supersaturated with mirabilite.

**Table 1:** Saturation Indices of Selected Minerals

	T°C	CaSO_4_ Anhydrite	CaCO_3_ Aragonite	BaSO_4_ Barite	CaCO_3_ Calcite	SrSO_4_ Celestite	CaSO_4_ ·2H_2_O Gypsum	MgCO_3_ Magnesite	Na_2_SO_4_ ·10H_2_O Mirabilite	SrCO_3_ Strontianite	BaCO_3_ Witherite
**Log K_sp_**		**-4.362**	**-8.336**	**-9.97**	**-8.406**	**-6.630**	**-4.581**	**-7.834**	**-1.214**	**-9.271**	**-8.652**

Ceylon Lake	20	-1.30	-1.03	*0.09*	-0.83	-0.30	-1.08	1.22	-0.73	-1.24	-4.97
	
	0	-1.31	-1.43	0.59	-1.22	-0.17	-1.11	0.96	0.25	-1.29	-4.98

Deadmoose Lake	20	-1.44	*-0.08*	0.28	0.12	-1.32	-1.20	1.25	-2.74	-1.17	-3.69
	
	0	-1.33	-0.37	0.73	-0.17	-1.23	-1.10	0.97	-1.69	-1.28	-3.76

Freefight Lake	20	-2.01	-0.67	*0.02*	-0.48	-2.35	-1.77	2.02	-1.54	-2.24	-3.98
	
	0	-1.95	-1.03	0.50	-0.82	-2.24	-1.74	1.74	-0.52	-2.32	-4.01

Little Manitou Lake	20	-0.93	*-0.09*	0.62	0.10	-0.62	-0.70	1.57	-2.12	-0.99	-3.88
	
	0	-0.85	-0.41	1.07	-0.21	-0.52	-0.64	1.29	-1.09	-1.09	-3.93

Waldsea Lake	20	-0.84	0.63	0.55	0.82	-0.71	-0.6	1.85	-2.71	-0.46	-3.31
	
	0	-0.75	0.33	1.01	0.54	-0.62	-0.52	1.59	-1.66	-0.54	-3.36

Mono Lake	20	-6.49	0.96	-3.03	1.15	ND	-6.24	1.20	-10.11	ND	-0.92
	
	0	-6.25	0.94	-2.40	1.14	ND	-6.03	1.23	-8.84	ND	-0.66

Pyramid Lake	20	-3.73	0.11	*-0.01*	0.30	-2.71	-3.48	1.45	-5.14	*-0.09*	-1.50
	
	0	-3.54	*-0.09*	0.49	0.12	-2.59	-3.31	1.23	-4.08	-0.14	-1.50

Walker Lake 5 m	20	-5.99	0.66	-1.73	0.85	-3.51	-5.74	1.92	-11.11	1.92	-0.42
	
	0	-5.71	0.57	-1.13	0.78	-3.27	-5.48	1.84	-9.99	2.01	-0.29

Abert Lake	20	ND	ND	-4.03	ND	ND	ND	ND	-10.73	ND	-0.93
	
	0	ND	ND	-3.41	ND	ND	ND	ND	-9.48	ND	-0.67

Goose Lake	20	-3.79	0.71	-0.90	0.90	-2.94	-3.54	0.43	-5.16	0.35	-1.73
	
	0	-3.53	0.62	-0.32	0.83	-2.71	-3.30	0.35	-4.04	0.44	-1.61

Lake Hoare 4 m		-3.16	-0.89	-1.40	-0.68	-3.18	-2.93	-1.58	-7.63	-1.92	-4.57

Lake Hoare 5 m		-2.85	-0.75	-1.10	-0.54	-2.83	-2.62	-1.36	-7.07	-1.74	-4.44

Lake Hoare 6 m		-2.44	-0.44	-0.67	-0.24	-2.41	-2.20	-1.00	-6.31	-1.43	-4.12

Lake Hoare 8 m		-2.22	-0.39	-0.44	-0.18	-2.17	-1.99	-0.90	-5.88	-1.35	-4.05

Lake Hoare 10 m		-2.09	-0.61	-0.31	-0.41	-2.03	-1.85	-1.11	-5.61	-1.57	-4.28

Lake Hoare 12 m		-2.03	-0.68	-0.26	-0.47	-1.98	-1.80	-1.17	-5.51	-1.65	-4.36

Lake Hoare 14 m		-1.99	-0.92	-0.22	-0.72	-1.95	-1.75	-1.42	-5.42	-1.90	-4.60

Lake Hoare 16 m		-1.90	-1.04	-0.13	-0.83	-1.87	-1.67	-1.54	-5.27	-2.02	-4.71

Lake Hoare 18 m		-1.88	-1.18	-0.12	-0.98	-1.86	-1.65	-1.68	-5.25	-2.17	-4.87

Lake Hoare 20 m		-1.87	-1.22	-0.12	-1.01	-1.86	-1.64	-1.71	-5.22	-2.21	-4.91

Lake Hoare 22 m		-1.88	-1.26	-0.11	-1.06	-1.85	-1.64	-1.76	-5.21	-2.25	-4.95

Lake Hoare 25 m		-1.86	-1.32	-0.11	-1.12	-1.85	-1.63	-1.83	-5.20	-2.32	-5.02

Lake Hoare 30 m		-1.85	-1.40	*-0.09*	-1.19	-1.84	-1.62	-1.90	-5.17	-2.39	-5.08

Lake Fryxell 6 m		-2.66	-0.55	-1.19	-0.34	-2.70	-2.43	-0.92	-5.39	-1.59	-4.53

Lake Fryxell 7 m		-2.65	-0.40	-1.21	-0.19	-2.70	-2.42	-0.75	-5.38	-1.46	-4.40

Lake Fryxell 8 m		-2.42	-0.27	-1.08	-0.07	-2.45	-2.18	-0.57	-4.86	-1.34	-4.38

Lake Fryxell 9 m		-2.42	-0.44	-0.85	-0.24	-2.40	-2.18	-0.57	-4.46	-1.46	-4.32

Lake Fryxell 10 m		-2.40	-0.46	-0.72	-0.25	-2.34	-2.16	-0.52	-4.31	-1.43	-4.22

Lake Fryxell 11 m		-2.41	-0.35	-0.62	-0.14	-2.35	-2.16	-0.33	-4.10	-1.33	-4.00

Lake Fryxell 12 m		-2.43	-0.29	-0.69	*-0.09*	-2.41	-2.19	-0.24	-4.11	-1.32	-4.00

Lake Fryxell 15 m		-2.46	-0.15	-0.64	*0.06*	-2.43	-2.22	*0.01*	-3.79	-1.16	-3.78

Lake Fryxell 18 m		-2.71	-0.14	-0.83	*0.07*	-2.67	-2.47	0.11	-3.87	-1.14	-3.70

Lake Vanda 10 m		-2.57	-0.82	-1.12	-0.62	-3.05	-2.31	-1.58	-7.15	-2.38	-4.83

Lake Vanda 60 m		-1.42	-0.62	-0.25	-0.42	-1.69	-1.15	-1.30	-5.37	-2.07	-4.87

Lake Vanda 70 m		-0.60	-0.75	*-0.09*	-0.56	-1.17	-0.40	-1.34	-4.43	-2.54	-5.56

East Lake Bonney 6 m		-1.82	-0.44	-0.25	-0.23	-1.96	-1.58	-0.70	-4.51	-1.62	-4.32

East Lake Bonney 22 m		-0.58	-0.89	0.59	-0.68	-0.42	-0.47	0.37	-1.42	-1.76	-5.16

East Lake Bonney 35 m		-0.26	-0.25	0.49	*-0.04*	*-0.06*	-0.28	1.25	-1.69	-1.14	-4.94

West Lake Bonney 5 m		-1.94	-0.47	-0.56	-0.27	-2.19	-1.70	-0.77	-4.72	-1.75	-4.54

West Lake Bonney 15 m		-0.53	-1.01	0.99	-0.80	-0.30	-0.33	-0.59	-1.08	-1.79	-4.93

West Lake Bonney 35 m		-0.52	-1.07	0.63	-0.86	-0.36	-0.36	-0.49	-0.81	-1.93	-5.36

Don Juan Pond		-0.37	ND	-1.69	ND	-1.25	-1.26	ND	-9.61	ND	ND

All of the lakes sampled in the Great Basin were supersaturated with respect to calcite and magnesite at 0°C and 20°C (calculations for Abert Lake were not attempted due to the undetectable Ca concentrations). All lakes except Pyramid Lake were supersaturated with aragonite at 0°C and 20°C. Pyramid Lake was supersaturated with aragonite at 20°C, but it was in approximate equilibrium at 0°C. Pyramid Lake was also near equilibrium with respect to barite at 20°C. Walker and Goose Lakes were supersaturated with strontianite at 0°C and 20°C, and strontianite was in near equilibrium at 20°C in Pyramid Lake.

**Figure 8:**
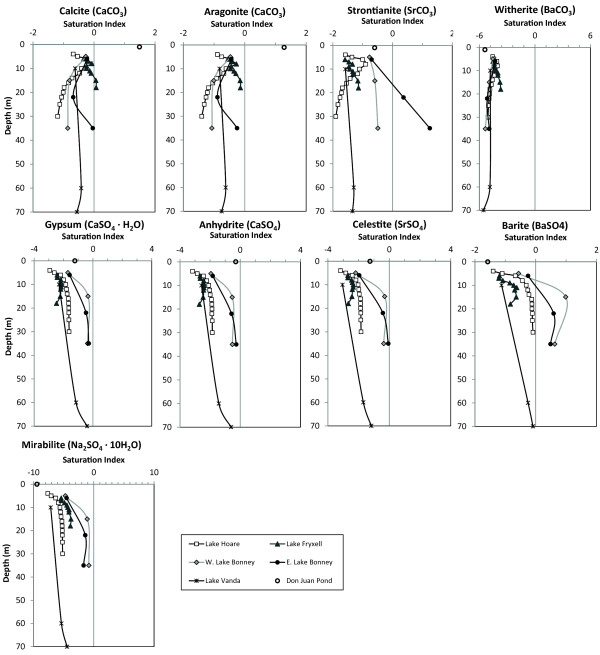
**Saturation indicies of selected minerals in the McMurdo Dry Valley Lakes**.

The lakes in the McMurdo Dry Valleys have been sampled in such a way that their saturation indices in profile could be examined at *in-situ *temperatures (Table 1). Lake Hoare was in near equilibrium with barite at 30 m. Calcite was in near equilibrium in Lake Fryxell at 8 m and from 12 to 14 m, and barite approached saturation at the sediment-water interface. Barite was in near equilibrium in the bottom water of Lake Vanda. In East Lake Bonney, barite and magnesite were supersaturated only at 22 and 35 m, and calcite and celestite were in near equilibrium at 35 m. In West Lake Bonney, barite was supersaturated at only 15 and 35 m. The profiles of the saturation indices of the various minerals are show in Figure 8.

## Discussion

Because chloride is conservative in most saline lakes, normalizing solute concentration relative to chloride can be used to monitor the progression of evaporation and chemical evolution [2]. Li and Rb show strong positive relationships with Cl indicating that their behaviour is conservative during brine evolution (Figures 3 and 4). These results suggest that the concentration of Li and Rb in all these closed basin lakes is determined by the rock-water interactions in source waters followed by evapoconcentration and that little removal has occurred at the salinities encountered in these lakes. This has previously been argued for Li in the Antarctic lakes by Lyons and Welch [20]. The McMurdo Valley lakes, in part because there are more cation data, show more richness in their trends. For example, in some samples Rb shows significant depletion relative to the trend with Cl at the lowest concentrations (Figure 4). This may reflect the dominant rock type in tillage in the watershed and a difference in source. Both Sr and Ba have depletions at intermediate Cl concentrations; the slope of the Ba vs. Cl changes little, however the Sr to Cl slope does change (Figure 5 and 7). These trends suggest removal of these alkaline earths at these intermediate chloride concentrations (~200 mg/L) and continued removal of Sr at even higher Cl concentrations.

### Binary salt precipitation

**Figure 9:**
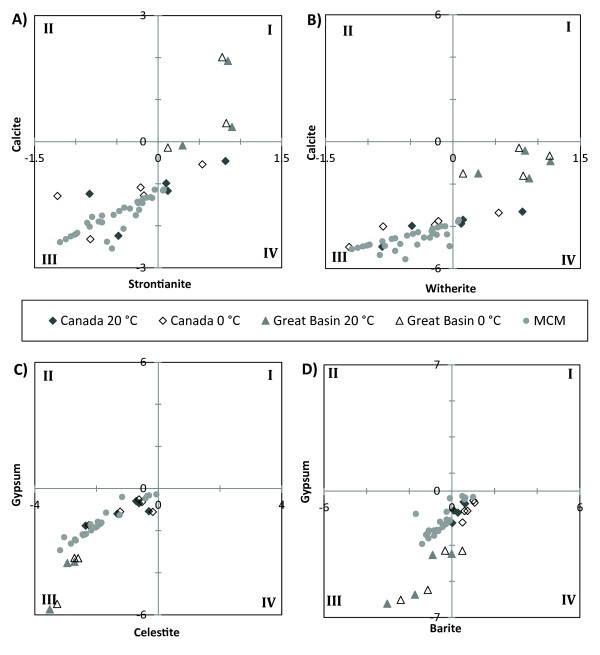
**The saturation indices of selected minerals in the lakes of this study**. A) Quadrant I: calcite and strontianite are supersaturated. Quadrant II: strontianite is supersaturated, and calcite is undersaturated. Quadrant III: strontianite and calcite are undersaturated. Quadrant IV: calcite is supersaturated, and strontianite is undersaturated. B) Quadrant I: calcite and witherite are supersaturated. Quadrant II: witherite is supersaturated, and calcite is undersaturated. Quadrant III: witherite and calcite are undersaturated. Quadrant IV: calcite is supersaturated, and witherite is undersaturated. C) Quadrant I: gypsum and celestite are supersaturated.

Strontium shows a poor relationship with Cl (Figure 5) in the sulphate and carbonate lakes, indicating that strontium does not behave conservatively and is removed from solution during lake water evolution. In Pyramid Lake, Ca, CO_3_, Sr, and Ba co-vary in deposited lake sediments suggesting a similar removal mechanism [21]. Strontium could form strontianite (SrCO_3_) or celestite (SrSO_4_) or be incorporated into CaCO_3_, specifically aragonite. Because the Canadian lakes remain undersaturated with respect to strontianite at both 0° and 20°C, it is unlikely that Sr is being removed as either of these minerals (Figure 9). Likewise, data from the McMurdo Dry Valley lakes indicate that the conditions for strontianite precipitation are thermodynamically unfavourable (Figure 8). Walker and Goose Lakes, however, are both supersaturated with respect to strontianite at 0°C and 20°C, and Pyramid Lake is in near equilibrium with strontianite at 0°C. It is therefore possible that Sr is removed in Walker and Goose Lakes by strontianite precipitation.

Celestite has been shown to be supersaturated in a hypersaline brine in the Mediterranean Sea and is thought to be a primary Sr removal mechanism there [22,23]. Celestite has been observed in the playa sediments of Bristol Dry Lake, California from the evaporation of saline groundwater discharge, and it is thought to be diagenetic in origin [24]. Celestite can also be produced diagenetically in part through the dolomitization of carbonate minerals [25]. Our work did not evaluate the lake sediments but was focused on the potential formation of primary, not diagenectically, produced celestite. Ba can also be removed via the diagenetic formation of celestite [25]. In all of the lakes examined in this work, celestite is undersaturated, the only exception being the bottom waters of East Lake Bonney where it is in near equilibrium (Figure 8). This does not rule out the possibility that celestite is forming diagenectically in these systems. Usually the depletion of Ba and Sr is coupled with the Ca depletion in a brine, and a continuous series of mineral precipitation from barite to celestite can exist [26,27]. In addition to being a removal "sink" for Ba, barite crystals in the ocean contain various amounts of Sr, that may exceed 20% [28].

The poor relationship of Ba to Cl at higher salinities indicates that barium is being removed in some of the lakes (Figure 7). Furthermore, our thermodynamic modelling shows that barite is supersaturated in some of the lakes investigated (Figure 9). Barite has a relatively low solubility and is supersaturated in surface waters such as the Mediterranean Sea and the brine-seawater interface of the Orca Basin in the Gulf of Mexico [22,23,29]. In the open ocean, Ba distributions are primarily influenced by primary production and the formation and dissolution of barite in the water column can lead to complex Ba concentration profiles [28,30-32]. In Saskatchewan, Deadmoose, Little Manitou, and Waldsea Lakes are supersaturated with respect to barite at 20°C, and Ceylon and Freefight Lakes are in near equilibrium at this temperature. However, at 0°C, all these lakes become supersaturated with respect to barite suggesting that a seasonal precipitation of barite may be an important control on the Ba concentration in the sulphate lakes (Table 1). Winter removal of barite may also be occurring in Pyramid Lake in the Great Basin as it is in equilibrium at 20°C but is supersaturated at 0°C (Table 1). The geochemical profiles of the McMurdo Dry Valley Lakes give insight into barite removal processes in stratified chloride-type brines (Figure 8). The fresher waters of Lake Hoare and Lake Vanda are undersaturated with respect to barite, but the lake waters approach equilibrium with depth. The surface waters of both lobes of Lake Bonney are undersaturated in barite but become supersaturated at and below the chemocline (Figure 8). These results suggest that inorganic precipitation of barite may be an important removal mechanism in both the chloride and sulphate lakes with higher TDS as the waters are evapoconcentrated.

Although the solubility of witherite (BaCO_3_) is low, it is rarely observed as a primary mineral, but rather as an alteration product of barite [26]. The calculations indicate that witherite is undersaturated in all lakes (Figure 9).

### Substitution

Clearly we are not in a position to evaluate the role of elemental substitution in this study as we have not analyzed the solid phase products. However, as noted above, previous work does suggest that this process could also affect the fate of these minor elements in salt lake systems.

Besides the precipitation of these minor alkali metals and alkaline earths via simple, binary salts as noted previously, the potential exists for their removal via substitution into more major element salts, such as CaCO_3 _and CaSO_4_·2H_2_O. The precipitation of calcite is the first geochemical divide encountered during most brine evolution [1]. The divalent elements Sr (K_d _= 0.182) and Ba (K_d _= 0.0195), have low K_d _values within calcite and therefore do not readily substitute into it [33]. However, these minerals may be more readily substituted for Ca in aragonite. It has also been demonstrated that inorganic aragonite, particularly at lower salinities, can accommodate approximately 4.5 times more Li than calcite [34]. Small amounts of Sr substitution can occur into gypsum and anhydrite, but this substitution is more pronounced during rapid precipitation [26].

With the exception of Abert Lake, which has no detectable alkalinity, the Great Basin lakes are supersaturated with respect to calcite, and the saturation indices are higher at 20°C than at 0°C. Walker, Mono, and Goose Lakes are supersaturated with aragonite at 0°C and 20°C, but Pyramid Lake only supersaturated at 20°C (Table 1). In Mono Lake, calcite and aragonite precipitate around calcium enriched springs discharging into the carbonate rich lake water [35]. In addition to aragonite and calcite, it has been noted that metastable ikaite (CaCO_3_·6H_2_O) precipitates along the shore during the winter months [36]. During the warmer months, ikaite is transformed into either CaCO_3 _or gaylussite (Na_2_Ca(CO_3_)2·5H_2_O) [37]. Sr and/or Ba may replace Ca in ikaite and be retained as it is transformed into calcite, aragonite, vaterite, or gaylussite.

Although currently undocumented, given the near freezing temperatures in the McMurdo Dry Valley lakes, ikaite precipitation could form and act as a mechanism for the removal of minor alkaline earth elements. Most occurrences of ikaite have been found in cold, anoxic marine sediments and it is also debatably linked to high orthophosphate concentrations [36,38]. Because orthophosphate was not included in the model calculations, we are unable to quantify the saturation indices of the waters in this study. However, previous work suggests that the MCM lakes are suitable environments for ikaite formation, especially since the PO_4 _concentration increases with depth in most of these lakes [39]. For example, the water below 9 meters in Lake Fryxell is anoxic, and dissolved phosphorous concentrations increase with depth in the lake [40]. These qualities in addition to temperatures circa 2°C at the bottom of Lake Fryxell should provide favorable conditions for ikaite formation. Additionally, in the deep, hypersaline waters of the ice-covered dry valley lakes, Vanda (in Wright Valley to the north), Bonney and Hoare may also present suitable environments for ikaite precipitation, as ikaite has been shown to form in hypersaline inclusions in Antarctic sea ice [41].

Our modelling suggests that the MCM lakes are undersaturated with respect to mirabilite (Table 1 and Figure 8). Mirabilite and gypsum have been shown to play a critical role in the evolution of Cl brines under freezing conditions [42,43]. In sub-zero conditions, mirabilite (Na_2_SO_4 _· 10H_2_O) is the first mineral to precipitate at -7.3°C [42]. The lowest temperature observed in the MCM lakes is -3.91°C in West Lake Bonney, indicating that the current temperatures in the MCM lakes perhaps are unfavorable for mirabilite precipitation. The McMurdo Dry Valley Lakes are predominately undersaturated with respect to calcite and aragonite, thus direct incorporation of Ba or Sr into aragonite or calcite crystals is unlikely. Calcium-sulphate minerals are also undersaturated, but in some instances, supersaturated with barite. All this suggests that Sr and Ba are not being removed by ionic substitution of calcium in calcite, aragonite, gypsum, or anhydrite.

The Canadian lakes in this study are mostly undersaturated with respect to calcite with the exceptions of Waldsea Lake (at 20°C and 0°C) and Little Manitou and Deadmoose Lakes at 20°C. This suggests that seasonal "whitings" of CaCO_3 _could be a potential sink for minor elements such as Sr. Aragonite has been attributed to whiting events in Waldsea and Deadmoose Lakes when the lakes are at moderate salinity and, during higher salinity periods, the lakes are capable of producing gypsum, aragonite, magnesite, and dolomite precipitates [44,45]. These calculations indicate that the removal of minor elements through the precipitation of gypsum, aragonite, and dolomite may vary spatially and temporally within these systems. The PHREEQ calculations indicate magnesite is supersaturated in all of the Saskatchewan lakes at high and low temperatures, but only Waldesa Lake is supersaturated with respect to aragonite (Table 1).

Due to their similar charges, ionic radii, and electronegativities, Rb can replace K in most crystal lattices. Evaporitic minerals such as carnallite and sylvite may incorporate Rb, but the precipitation of these salts is limited due to their very high solubility. For example, during the final stages of seawater evaporation, Rb preferentially concentrates in carnallite at concentrations of up to 300 ppm [46]. During these final stages, other highly soluble salts such as the chlorides and borates reach saturation. The solubility of lithium salts are so high that it concentrates beyond bischofite precipitation and may be incorporated in halite, polyhalite, carnallite, sylvites, and borates [26,47,48]. Lithium does also occur in halite and other last-precipitating Na and K rich salts. At some point during the end stages of evapoconcentration and halite precipitation, all strontium is removed from the brine as SrCl because the solubility of SrCl decreases dramatically in the presence of NaCl [26]. Although the lakes in this study are at various stages of brine evolution, there is no evidence to suggest that Rb or Li are being removed from the lakes as binary salts as all lakes in the study are grossly undersaturated with respect to halite, let alone the more soluble salts, carnallite and sylvite.

### The potential importance of biogenic precipitation

Celestite precipitation may occur inorganically or through biological activity. In freshwater systems, cyanobacteria have been shown to mediate the formation of celestite and strontianite [49]. Strontium depletion in the upper ocean has been ascribed to the production of a celestite skeleton by surface-dwelling acantharia [50]. The death and decomposition of these organisms can also facilitate in the precipitation of barite [51]. A strong correlation between barium and nutrient concentration in the surface water of the oceans suggests biotic control of Ba distribution in seawater [28,52]. Although barite production is common in the open ocean, it functions as more of a transport mechanism rather than a permanent sink of barium as approximately 85% is released into the deep ocean during the breakdown and mineralization of organic matter [53]. This dissolution of barite is also evidenced by a small barium concentration maximum at the O_2_/H_2_S interface in the Black Sea [30]. Strontium also has been shown to be influenced by biogeochemical processes in the marine environment and in some lacustrine environments. Although these ecosystems are quite different than the lakes in this study, biologically mediated transport cannot be ruled out as a process occurring in the systems studied here. Clearly more work needs to be done in the investigation of biological removal of these minor elements in salt lake systems.

## Conclusions

This study has elucidated potential controls on the concentrations of minor elements in a number of saline, closed-basin lakes. In all the anionic water types (i.e. carbonate, sulphate and chloride), lithium and rubidium appear to behave conservatively and are the result of solute input and evapoconcentration. Unlike this "conservative" behaviour of lithium and rubidium, strontium and barium do not behave conservatively. Thermodynamic calculations suggest they are removed by different mechanisms depending on brine type. Strontium appears to be depleted in sulphate and carbonate brines. In the carbonate brines of the Great Basin, Sr may be removed as strontianite (SrCO_3_) or substituted for Ca in CaCO_3 _minerals such as ikaite, or especially aragonite. Strontium may be removed from the sulphate-type lakes of Saskatchewan by substitution into CaCO_3 _as these lakes are supersaturated with respect to both calcite and aragonite. Gypsum is a phase that does not appear to be important for Sr removal in these lacustrine systems, as it is undersaturated in all of the lakes under investigation. Barite is supersaturated in the McMurdo Dry Valley lakes (chloride-type) and the Saskatchewan lakes indicating that it is a possible important sink for Ba in these systems. Barite and celestite have been shown to be biologically precipitated in marine systems, and the role of biogenic removal of barium and strontium in these saline lakes warrants further investigation.

## Methods

### Sample collection

Sample collection bottles for cations were cleaned by soaking the bottles in dilute HCl and rinsing them with 18 MΩ water (DI). Anion collection bottles were soaked and rinsed in DI. Stream samples were collected in pre-cleaned Nalgene polyethylene bottles after rinsing the bottles with sample water three times. For the Great Basin and Canadian lakes, only surface samples were collected while depth profiles are available for the Antarctic lakes. Samples were collected from the McMurdo Dry Valley lakes at various depths by lowering a Niskin bottle into a hole drilled into the ice and filling a pre-cleaned Nalgene polyethylene bottle [10]. Great Basin lake samples were collected in pre-cleaned polyethylene bottles. After collection, samples were stored in the dark and sent to The Ohio State University. All samples from Antarctica and the Great Basin were filtered through 0.4 μm Nuclepore filters and cation samples were preserved with trace-metal grade HNO_3 _within 1 week after collection. Samples from Saskatchewan were obtained from the archives at the University of Manitoba [54]. These samples were filtered through 0.4 μm Nuclepore filters and preserved with trace-metal grade HNO_3 _at The Ohio State University.

### Laboratory analysis

Samples and field blanks were analyzed for Cl, SO_4_, Na, K, Mg, and Ca on Dionex DX-120 Ion Chromatograph (IC) using modified techniques from [55]. Precision of our Cl and SO_4 _measurements were < 1 and 2%, respectively. An independent multi-element standard was analyzed after creating a calibration curve to satisfy quality assurance and quality control. In order to determine the reproducibility of this analytical method, selected samples were analyzed twice.

Li, Rb, Sr, and Ba were quantified using a Perkin-Elmer Sciex ELAN 6000 Inductively Coupled Plasma Mass spectrometer (ICPMS). An attempt was made to quantify Cs concentrations, however all sample were below the detection limit of 0.05 μg/L. In addition to sample analysis, the concentrations of Li, Rb, Sr, and Ba were quantified in field blanks (i.e. bottles filled with DI that were transported to the field and processed as samples). Multi-element standards were used for six and eight point calibration curves. Calibration curves were analyzed at least twice in an analytical run. For approximately half of the samples, instrument drift was monitored by using a mid-concentration check standard every 1 to 5 samples. The reproducibility of this standard is illustrated by the relative standard deviations of analyses. Precision of the Li, Rb, Sr and Ba lake water measurements were ± 1.8%, 0.7%, 0.7% and 0.7%, respectively. For the other samples, a 20 μg/L internal standard of Be, Co, La, and Y was implemented for the high salinity samples as a means of allowing the instrument to correct for drift.

Sample aliquots from the McMurdo Dry Valley lakes were analyzed for dissolved inorganic carbon (DIC). The samples were bottled with no head space and preserved with 0.5% v:v chloroform. The samples were stored chilled in the dark until analysis by either a Lira IRGA with an HP integrator or a Licor 6252 system in the Crary Lab at McMurdo Station, Antarctica [56]. Because the lakes are stratified, DIC replicate measurements below the chemoclines can be compared to illustrate the precision of the method. Alkalinity for the Great Basin and Canadian lakes was analyzed by titration at The Ohio State University. This was achieved by titrating 10 mL sample aliquots with 1 N Optima HCl. The precision of this method has been shown to be within 5% [57].

### Geochemical modelling

Saturation indices were analysed by PHREEQCI, a graphical interface of the thermodynamic model PHREEQ [58,59]. Great Basin and Canadian lake indices were calculated at 20°C and 0°C, and MCM lake indices were calculated based on *in-situ *temperature measurements. PHREEQ is a general geochemical modelling program that is suitable for many different aqueous environments, but it is necessary to illustrate some of the program's limitations. The default program uses ion associations and Debye-Hückle equations to determine the saturation indices of a suite of minerals. Due to the high ionic strength of the solutions in this study, Debye-Hückle equations are not appropriate thus a modified Pitzer database was used. The modifications were the addition of thermodynamic data for reactions and species not included in the Pitzer database [58]. It should be noted that the initial database was a compilation of equilibrium constants and enthalpies from various sources [58]. The thermodynamic information used to modify the Pitzer database was gleaned from the *Minteq *database included in the PHREEQCI package [59]. Barite was not included in the *Minteq *database, so its thermodynamic data were tabulated from Stumm and Morgan [60].

## Competing interests

The authors declare that they have no competing interests.

## Authors' contributions

This manuscript is modified from a chapter in the Ph.D. dissertation of RW completed at The Ohio State University under the supervision of WBL. RW did the analysis of the minor elements and all the modelling work. WBL helped conceive of the study and participated in the design and coordination of these investigations as well as helped develop the manuscript in all its phases. All the authors have read and approved the final manuscript.

## Supplementary Material

Additional file 1 **Supplemental data**. Reactions, products and phases added to the Pitzer database in PHREEQ. Click here for file

Additional file 2 **Supplemental data**. Li, Rb, Sr, and Ba concentrations. Click here for file

Additional file 3 **Supplemental data**. Major ion, pH, alkalinity, silica and temperature data. Click here for file
