# Supplementary material for: The fate of minor alkali elements in the chemical evolution of salt lakes
Source: Saline Syst. 2011 Oct 12;7:2. doi: 10.1186/1746-1448-7-2 (PMC3213058; doi:10.1186/1746-1448-7-2)
Supplement: Additional file 1 — Supplemental data. Reactions, products and phases added to the Pitzer database in PHREEQ. [file 1746-1448-7-2-S1.DOCX]

**Additional file 1 – Supplemental data**

Major ion, pH, alkalinity, silica and temperature data.

|  | **F**  **mg/l** | **Cl**  **mg/l** | **Br**  **mg/l** | **SO_4_**  **mg/l** | **Na**  **mg/l** | **K**  **mg/l** | **Mg**  **mg/l** | **Ca**  **mg/l** | **pH** | **alk**  **mg C/l** | **Si**  **mg/l** | **T**  **°C** |
| --- | --- | --- | --- | --- | --- | --- | --- | --- | --- | --- | --- | --- |
| ***Saskatchewan Lakes*** |  |  |  |  |  |  |  |  |  |  |  |  |
| Ceylon L. | DL | 6,980 | 100 | 196,000 | 23,500 | 2,600 | 2,320 | 33 | 7.88 | 850 | nd | nd |
| Deadmoose L. | 11 | 8,160 | 8 | 14,800 | 2,240 | 4,20 | 525 | 34 | 8.35 | 730 | nd | nd |
| Freefight L. | DL | 4,190 | 43 | 92,600 | 8,750 | 2,100 | 2,140 | 7.9 | 7.91 | 6,000 | nd | nd |
| Little Manitou L. | DL | 21,300 | 93 | 51,100 | 4,460 | 910 | 2,080 | 85 | 8.04 | 850 | nd | nd |
| Waldsea L. | 1.0 | 8,160 | DL | 30,200 | 2,160 | 610 | 1,320 | 120 | 8.54 | 1,049 | nd | nd |
| ***Great Basin Lakes*** |  |  |  |  |  |  |  |  |  |  |  |  |
| Mono L. | 55 | 16,300 | 15 | 8,970 | 33,700 | 1,500 | 6.24 | 3.7 | 9.87 | 32,000 | nd | nd |
| Pyramid L. | 1.5 | 1,840 | 1.7 | 276 | 459 | 94 | 31.9 | 1.3 | 9.22 | 1,300 | nd | nd |
| Walker L. 5m | 22 | 3,580 | 3.1 | 3,430 | 6,350 | 260 | 40.3 | 2.2 | 9.52 | 4,400 | nd | nd |
| Abert L. | 3.8 | 29,800 | 73 | 1,370 | 51,500 | 1,400 | DL | DL | 9.88 | 37,000 | nd | nd |
| Goose L. | 0.92 | 602 | DL | 307 | 467 | 96 | 1.31 | 2.5 | 9.70 | 2,900 | nd | nd |
| ***McMurdo Dry Valley Lakes*** |  |  |  |  |  |  |  |  |  |  |  |  |
| Lake Hoare 4m | 0.29 | 33.5 | DL | 13.5 | 20.3 | 3.6 | 3.64 | 13 | 9.05 | 10.4 | 1.2 | 0.00 |
| Lake Hoare 5m | 0.26 | 33.7 | DL | 17.9 | 24.0 | 3.8 | 4.55 | 13 | 8.92 | 10.3 | 1.1 | 0.32 |
| Lake Hoare 6m | 0.63 | 73.6 | DL | 38.6 | 54.1 | 8.9 | 10.1 | 27 | 8.85 | 22.6 | 2.2 | 0.57 |
| Lake Hoare 8m | 1.1 | 116 | DL | 62.3 | 90.6 | 15.0 | 16.8 | 40 | 8.70 | 36.6 | 3.4 | 0.40 |
| Lake Hoare 10m | 1.3 | 147 | DL | 77.2 | 112 | 18.7 | 21.1 | 49 | 8.42 | 47.6 | 5.1 | 0.47 |
| Lake Hoare 12m | 1.3 | 152 | DL | 79.4 | 120 | 20.0 | 22.3 | 51 | 8.14 | 51.4 | 5.3 | 0.55 |
| Lake Hoare 14m | 1.5 | 176 | DL | 90.3 | 137 | 22.8 | 25.0 | 58 | 7.85 | 60.1 | 5.8 | 0.45 |
| Lake Hoare 16m | 1.6 | 186 | DL | 95.1 | 145 | 24.0 | 26.8 | 64 | 7.64 | 67.2 | 6.3 | 0.26 |
| Lake Hoare 18m | 1.7 | 195 | DL | 99.3 | 154 | 25.4 | 28.3 | 67 | 7.54 | 73.5 | 6.7 | 0.24 |
| Lake Hoare 20m | 1.7 | 200 | DL | 102 | 156 | 25.9 | 28.9 | 68 | 7.46 | 76.1 | 6.6 | 0.24 |
| Lake Hoare 22m | 1.7 | 200 | DL | 101 | 157 | 25.7 | 28.6 | 68 | 7.37 | 77.5 | 6.7 | 0.24 |
| Lake Hoare 25m | 1.8 | 207 | DL | 105 | 160 | 26.6 | 29.6 | 70 | 7.34 | 79.4 | 6.4 | 0.20 |
| Lake Hoare 30m | 1.7 | 206 | DL | 104 | 165 | 27.4 | 30.4 | 72 | 7.28 | 86.5 | 7.0 | 0.17 |
| Lake Fryxell 6m | 1.1 | 416 | DL | 62.1 | 296 | 29.4 | 34.8 | 59 | 7.95 | 96 | 4.4 | -0.03 |
| Lake Fryxell 7m | 1.2 | 429 | DL | 63.4 | 311 | 30.6 | 37.5 | 61 | 7.95 | 130 | 4.7 | 0.44 |
| Lake Fryxell 8m | 1.7 | 728 | DL | 102 | 532 | 47.8 | 61.5 | 89 | 7.86 | 164 | 6.8 | 1.33 |
| Lake Fryxell 9m | 2.0 | 1,090 | DL | 124 | 850 | 68.6 | 97.9 | 94 | 7.54 | 259 | 9.4 | 1.55 |
| Lake Fryxell 10m | 2.4 | 1,330 | DL | 144 | 980 | 77.8 | 110 | 93 | 7.50 | 296 | 10.6 | 1.69 |
| Lake Fryxell 11m | 3.8 | 1,710 | DL | 156 | 1,300 | 101 | 146 | 100 | 7.48 | 403 | 12.8 | 1.84 |
| Lake Fryxell 12m | 3.2 | 1,850 | DL | 152 | 1,330 | 104 | 158 | 100 | 7.53 | 407 | 12.1 | 1.91 |
| Lake Fryxell 15m | 5.3 | 2,890 | DL | 163 | 2,160 | 158 | 248 | 130 | 7.50 | 591 | 17.3 | 2.06 |
| Lake Fryxell 18m | 5.5 | 3,680 | DL | 105 | 2,680 | 186 | 319 | 130 | 7.46 | 694 | 16.8 | 2.14 |
| E. Lake Bonney 6m | DL | 679 | DL | 158 | 354 | 13.1 | 51.3 | 72 | 8.71 | 19.4 | nd | 1.57 |
| E. Lake Bonney 22m | DL | 111,000 | DL | 3,010 | 27,700 | 1,480 | 21,400 | 1,400 | 6.05 | 230 | nd | 4.69 |
| E. Lake Bonney 35m | DL | 160,000 | DL | 3,170 | 48,500 | 2,780 | 31,500 | 1,600 | 6.76 | 54.1 | nd | 0.96 |
| W. Lake Bonney 5m | DL | 548 | DL | 124 | 286 | 9.27 | 39 | 59 | 8.75 | 16.2 | nd | 0.85 |
| W. Lake Bonney 17m | DL | 51,300 | DL | 3,750 | 23,300 | 701 | 5,360 | 1,800 | 6.07 | 513 | nd | 0.14 |
| W. Lake Bonney 37m | DL | 86,800 | DL | 4,600 | 39,700 | 1,290 | 9,180 | 2,300 | 5.74 | 771 | nd | -3.91 |
| Lake Vanda 10m | DL | 251 | DL | 16.9 | 52.7 | 12.2 | 19.5 | 81 | 8.35 | 10.3 | nd | 4.20 |
| Lake Vanda 62m | DL | 20,400 | DL | 194 | 1,710 | 152 | 2,000 | 6,900 | 6.72 | 29.7 | nd | 15.03 |
| Lake Vanda 70m | DL | 58,900 | DL | 525 | 5,000 | 451 | 6,470 | 21,000 | 5.91 | 68.8 | nd | 21.09 |
| Don Juan Pond | 12 | 283,000 | DL | 142 | 12,200 | 150 | 2,000 | 140,000 | 6.15 | nd | nd | nd |
| **Seawater (Pilson, 1998)** | 1.3 | 19,830 | 69.0 | 2,779 | 11,048 | 409 | 1,316 | 422.1 | 8.14** | 129* |  |  |
