# Supplementary material for: The fate of minor alkali elements in the chemical evolution of salt lakes
Source: Saline Syst. 2011 Oct 12;7:2. doi: 10.1186/1746-1448-7-2 (PMC3213058; doi:10.1186/1746-1448-7-2)
Supplement: Additional file 2 — Supplemental data. Li, Rb, Sr, and Ba concentrations. [file 1746-1448-7-2-S2.DOCX]

**Additional file 2 – Supplemental data**

Li, Rb, Sr, and Ba concentrations.

|  | **Li**  **µg/l** | **Rb**  **µg/l** | **Sr**  **µg/l** | **Ba**  **µg/l** |
| --- | --- | --- | --- | --- |
| ***Saskatchewan Lakes*** |  |  |  |  |
| Ceylon Lake | 13,100 | 98.8 | 3,180 | 40 |
| Deadmoose Lake | 1,900 | 20.2 | 490 | 16 |
| Freefight Lake | 5,700 | 70.3 | 35.1 | 20 |
| Little Manitou Lake | 11,400 | 37.4 | 1,890 | 54 |
| Waldsea Lake | 2,900 | 25.1 | 1,800 | 38 |
| ***Great Basin Lakes*** |  |  |  |  |
| Mono Lake | 9,430 | 1630 | DL | 40 |
| Pyramid Lake | 465 | 34.4 | 126 | 40 |
| Walker Lake 5m | 1,390 | 31.7 | 4,850 | 160 |
| Abert Lake | 67.2 | 261 | DL | 38 |
| Goose Lake | 5.90 | 8.60 | 132 | 8 |
| ***McMurdo Dry Valleys Lakes*** |  |  |  |  |
| Lake Hoare 4m | 2.47 | 2.11 | 150 | 2 |
| Lake Hoare 5m | 4.06 | 2.16 | 228 | 3 |
| Lake Hoare 6m | 7.06 | 3.31 | 389 | 5 |
| Lake Hoare 8m | 9.75 | 4.37 | 549 | 7 |
| Lake Hoare 10m | 12.3 | 5.24 | 669 | 9 |
| Lake Hoare 12m | 13.2 | 5.55 | 714 | 10 |
| Lake Hoare 14m | 14.1 | 5.67 | 774 | 10 |
| Lake Hoare 16m | 15.9 | 5.96 | 865 | 12 |
| Lake Hoare 18m | 16.6 | 5.96 | 878 | 12 |
| Lake Hoare 20m | 16.1 | 5.90 | 883 | 12 |
| Lake Hoare 22m | 16.3 | 5.97 | 893 | 12 |
| Lake Hoare 25m | 16.5 | 6.01 | 904 | 12 |
| Lake Hoare 30m | 16.8 | 5.98 | 928 | 13 |
| Lake Fryxell 6m | 23.6 | 6.38 | 668 | 5 |
| Lake Fryxell 7m | 24.2 | 6.10 | 673 | 5 |
| Lake Fryxell 8m | 39.6 | 9.18 | 1,010 | 6 |
| Lake Fryxell 9m | 61.5 | 12.0 | 1,200 | 11 |
| Lake Fryxell 10m | 63.1 | 13.1 | 1,310 | 14 |
| Lake Fryxell 11m | 73.7 | 14.5 | 1,420 | 20 |
| Lake Fryxell 12m | 69.7 | 13.4 | 1,300 | 18 |
| Lake Fryxell 15m | 92.7 | 17.1 | 1,680 | 27 |
| Lake Fryxell 18m | 99.1 | 19.6 | 1,770 | 33 |
| E. Lake Bonney 6m | 29.3 | 2.80 | 624 | 9 |
| E. Lake Bonney 22m | 4,900 | 150 | 32,500 | 150 |
| E. Lake Bonney 35m | 8,290 | 294 | 45,100 | 140 |
| W. Lake Bonney 5m | 22.4 | 1.50 | 399 | 4 |
| W. Lake Bonney 17m | 2,810 | 96.1 | 36,500 | 240 |
| W. Lake Bonney 37m | 4,400 | 156 | 50,400 | 200 |
| Lake Vanda 10m | 114 | 2.50 | 308 | 8 |
| Lake Vanda 62m | 7,740 | 37.0 | 18,600 | 230 |
| Lake Vanda 70m | 22,000 | 94.3 | 69,300 | 660 |
| Don Juan Pond | 265,000 | 102 | 1,040,000 | 1,400 |
| Seawater (Pilson, 1998) | 178 | 120 | 8,130 | 14.1 |
