# Supplementary material for: The fate of minor alkali elements in the chemical evolution of salt lakes
Source: Saline Syst. 2011 Oct 12;7:2. doi: 10.1186/1746-1448-7-2 (PMC3213058; doi:10.1186/1746-1448-7-2)
Supplement: Additional file 3 — Supplemental data. Major ion, pH, alkalinity, silica and temperature data. [file 1746-1448-7-2-S3.DOCX]

**Additional file 3 – Supplemental data**

Reactions, products and phases added to the Pitzer database in PHREEQ.

| ***Reactions:*** | ***Products:*** | ***Phases:*** |
| --- | --- | --- |
| H_4_SiO_4_ = H_4_SiO_4_ | SrOH | Strontianite: SrCO_3_ |
| H_4_SiO_4_ = H_3_SiO_4_^-^ + H^+^ | SrHCO_3_ | Witherite: BaCO_3_ |
| H_4_SiO_4_ = H_2_SiO_4_^2-^ + 2H^+^ | SrCO_3_ | Amorphous Silica: SiO_2_(a) |
| CO_3_^2-^ + 10H^+^ + 8e^-^ = CH_4_ + H_2_O | SrSO_4_ | Calcedony: SiO_2_ |
| Ca^2+^ + H_2_O = CaOH + H^+^ | LiOH | Quartz: SiO_2_ |
| Ca^2+^ + CO_3_^2-^ + H^+^ = CaHCO_3_^+^ | LiSO_4_ | Sepiolite (d): Mg_4_Si_6_O_15_(OH)_2_·6(H_2_O) |
| Ca^2+^ + HSO_4_^-^ = CaHSO_4_^+^ | SiF_6_^2-^ | Sepiolite: Mg_4_Si_6_O_15_(OH)_2_·6(H_2_O) |
| Ba^2+^ + H_2_O = BaOH^-^ + H^+^ | MgF^+^ | BaF_2_ |
| Ba^2+^ + CO_3_^2-^ = BaCO_3_ | CaF^+^ | SrF_2_ |
| Ba^2+^ + HCO_3_^-^ = BaHCO_3_^+^ | NaF | Barite: BaSO_4_ |
| Ba^2+^ + SO_4_^2-^ = BaSO_4_ | HF |  |
| Rb^+^ = Rb^+^ | HF^2-^ |  |
| F^-^ = F^-^ | H_2_F_2_ |  |
